# COVID-19 cases presenting to the Emergency Department predict Qatar National COVID-19 trends and numbers

**DOI:** 10.5339/qmj.2021.56

**Published:** 2021-10-21

**Authors:** Sameer A. Pathan, Jibin Moinudheen, Katie Simon, Stephen H. Thomas

**Affiliations:** ^1^Corporate Department of Emergency Medicine, Hamad Medical Corporation, Qatar E-mail: SPathan@hamad.qa; ^2^Blizard Institute, Barts and The London School of Medicine, Queen Mary Univ. of London, UK; ^3^School of Public Health and Preventive Medicine, Monash University, Melbourne, Australia

**Keywords:** COVID-19, prediction, national trends, emergency medicine

## Abstract

In this short communication, we summarized the analyses, models, and interpretations of the corporate department of emergency medicine's (CDEM) COVID-19 numbers and their relationship to predict the national COVID-19 trends and numbers in Qatar. Data included in this analysis were obtained between March 1, 2020 and July 31, 2021. It included the number of COVID-19 cases that presented to four major EDs under the Hamad Medical Corporation CDEM umbrella and published data from the Qatar Ministry of public health (MoPH). On plotting weighted scatterplot smoothing (lowess) trend lines, there were striking similarities between CDEM and national COVID-19 *n* curves for overall trends and peaks. In conclusion, CDEM COVID-19 spike may be useful to predict national COVID-19 spike in 2–3 weeks.

The health and socioeconomic impacts of the COVID-19 pandemic were observed worldwide. Many government and public authorities have tried and developed various models to predict nationwide COVID-19 wave patterns and numbers.^1^ However, little has been published about predicting COVID-19 trends in the State of Qatar.^2,3^ The Hamad Medical Corporation (HMC) is the principal government-funded healthcare provider in the State of Qatar.

Most COVID-19 cases in Qatar were adults. They presented for medical assessment to the HMC's Corporate Department of Emergency Medicine (CDEM), which operates through four emergency departments strategically located to cover the geography of Qatar's habitation. Qatar's confirmation of COVID-19 positivity is through a real-time reverse transcription-polymerase chain reaction (rRT-PCR) test. Qatar's number of new cases is published daily on the Ministry of public health (MoPH) website.^4^

In May 2020, the HMC's CDEM Research Division noted that the national peak occurred approximately 2.5 weeks after peak COVID-positivity was noted in the HMC EDs. The pattern of approximately 2.5 weeks’ interval between CDEM and national peaks also occurred in the Spring 2021 Qatari COVID peak. Plots of the daily CDEM COVID and national COVID *numbers* are combined in Figure 1.

Figure 1 depicts that for the May 2020 national COVID peak, there was an interval of 18 days between the HMC ED peak and the nationwide peak. Figure 1 also shows the 19-day period between the CDEM peak and national peak that was seen in the April/May 2021 time frame.

The striking similarity of the CDEM and national COVID *n* curves is emphasized when the national curve is “frame-shifted” 18 days to the left on the *x*-axis. In Figure 2, a given “index” day's CDEM COVID *n* is plotted paired (*i.e.,* in vertical alignment) with the nationwide COVID *number* for 18 days after the index date. In Figure 2, the locally-weighted scatterplot smoothing (lowess) trend depicts a relationship between variables and foresees trends using the smooth line, lines to emphasize plots’ similarities in overall trends and peaks.

A simple regression model was generated to assess the precision of the predictive value of CDEM COVID diagnoses to forecast the national COVID numbers that would be seen 18 days later. The model demonstrated that simply knowing the CDEM COVID volume on a given day could predict national COVID levels in 2–3 weeks, with 90% accuracy (*r* ^2^.88, *p* < .001). The coefficient for ED prediction of national COVID *n* was 3.9 (95% CI based on Huber–White sandwich method: 3.7–4.1).

An overall plot of model-predicted *vs.* actual national COVID numbers in shown in Figure 3. The figure shows that the ED case numbers tend to underestimate the national COVID numbers but also that the overall trends are similar. The lowess trend line for model-predicted *vs.* actually reported national COVID *n* is close to the 45-degree line of perfect prediction.

While Figure 3 demonstrates that the ED-based prediction tends toward occasional underestimation of national numbers, Figure 4 demonstrates the overall “fit” of the predicted *vs.* actual Qatar-wide COVID data (the green 45-degree line represents perfect fit and the model's lowess trend is the orange dashed line).

We conclude that for COVID, the occurrence of ED peaks should be interpreted as predicting a likely national peak within 2–3 weeks. Furthermore, we found that the reasonably rapid resolution of the ED COVID numbers reliably predicts the national-level resolution within 2–3 weeks.

## Figures and Tables

**Figure 1.**
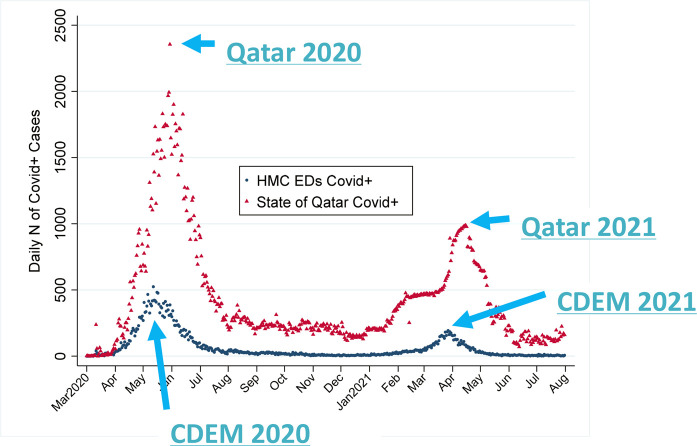
CDEM and nationwide daily newly diagnosed COVID-19 cases

**Figure 2.**
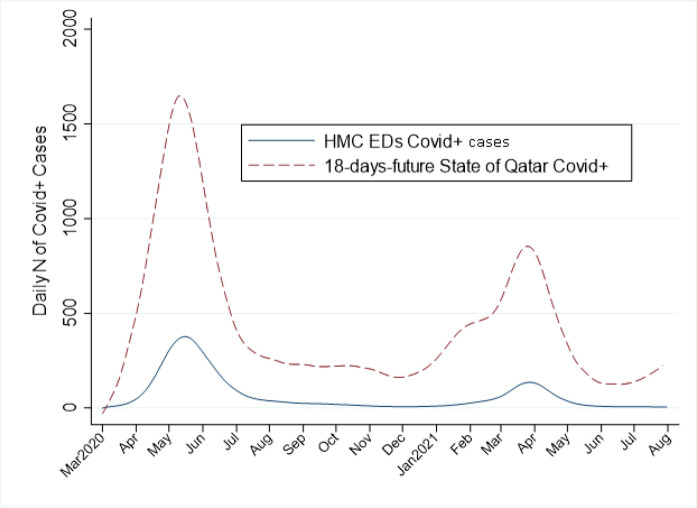
CDEM daily COVID-19 cases and national new cases in 18 days’ time

**Figure 3.**
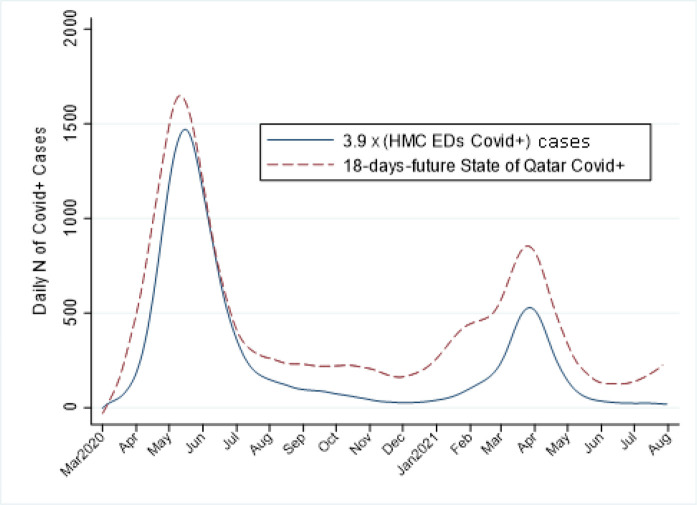
National COVID-positive diagnoses- Predictions from ED-based model vs. actual

**Figure 4.**
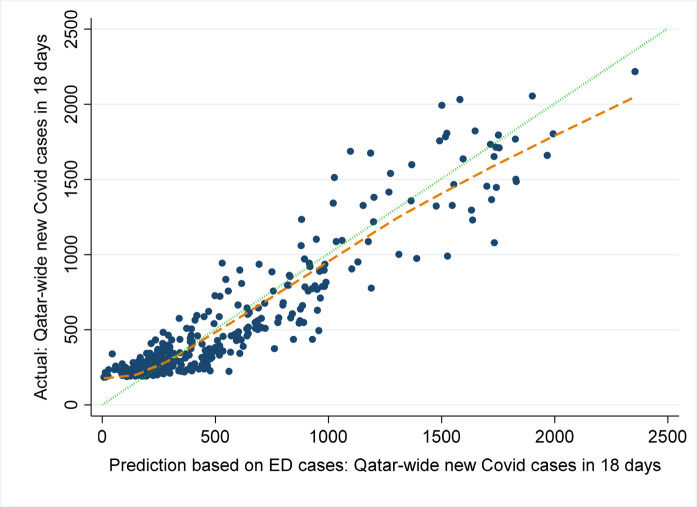
Scatterplot of ED-based model-predicted and actual national COVID n
